# Electrochemotherapy for head and neck cancers: possibilities and limitations

**DOI:** 10.3389/fonc.2024.1353800

**Published:** 2024-02-15

**Authors:** Arnoldas Morozas, Veronika Malyško-Ptašinskė, Julita Kulbacka, Justinas Ivaška, Tatjana Ivaškienė, Vitalij Novickij

**Affiliations:** ^1^ Department of Immunology and Bioelectrochemistry, State Research Institute Centre of Innovative Medicine, Vilnius, Lithuania; ^2^ Faculty of Electronics, Vilnius Gediminas Technical University, Vilnius, Lithuania; ^3^ Department of Molecular and Cellular Biology, Wroclaw Medical University, Wroclaw, Poland; ^4^ Faculty of Medicine, Vilnius University, Vilnius, Lithuania

**Keywords:** head and neck cancer, electrochemotherapy, tumors, pulsed electric fields, FEM analysis

## Abstract

Head and neck cancer continues to be among the most prevalent types of cancer globally, yet it can be managed with appropriate treatment approaches. Presently, chemotherapy and radiotherapy stand as the primary treatment modalities for various groups and regions affected by head and neck cancer. Nonetheless, these treatments are linked to adverse side effects in patients. Moreover, due to tumor resistance to multiple drugs (both intrinsic and extrinsic) and radiotherapy, along with numerous other factors, recurrences or metastases often occur. Electrochemotherapy (ECT) emerges as a clinically proven alternative that offers high efficacy, localized effect, and diminished negative factors. Electrochemotherapy involves the treatment of solid tumors by combining a non-permeable cytotoxic drug, such as bleomycin, with a locally administered pulsed electric field (PEF). It is crucial to employ this method effectively by utilizing optimal PEF protocols and drugs at concentrations that do not possess inherent cytotoxic properties. This review emphasizes an examination of diverse clinical practices of ECT concerning head and neck cancer. It specifically delves into the treatment procedure, the choice of anti-cancer drugs, pre-treatment planning, PEF protocols, and electroporation electrodes as well as the efficacy of tumor response to the treatment and encountered obstacles. We have also highlighted the significance of assessing the spatial electric field distribution in both tumor and adjacent tissues prior to treatment as it plays a pivotal role in determining treatment success. Finally, we compare the ECT methodology to conventional treatments to highlight the potential for improvement and to facilitate popularization of the technique in the area of head and neck cancers where it is not widespread yet while it is not the case with other cancer types.

## 1 Introduction

The incidence of head and neck cancer (HNC; including lip/oral cavity, larynx, oropharynx, hypopharynx, salivary glands, and nasopharynx) in Europe is approximately 21.8 per 100,000, with a mortality rate of approximately 15.6 per 100,000 (1). Squamous cell carcinoma is the most common type of cancer in the HNC region; it accounts for more than 90% of the cases (2). Smoking and heavy alcohol consumption are still the main risk factors, and their joint effect increases the risk for cancer even more (3). Furthermore, the underlying causes of some of the sites in the head and neck (H&N) region are changing. There has been a constant rise in the incidence of oropharyngeal cancers due to increasing rates of human papillomavirus infection (4). Over a hundred varieties of human papillomavirus (HPV) have been identified. The most oncogenic types are 16 and 18, which account for more than 90% of cases of oropharyngeal cancer related to HPV (5).

**Figure 1:**
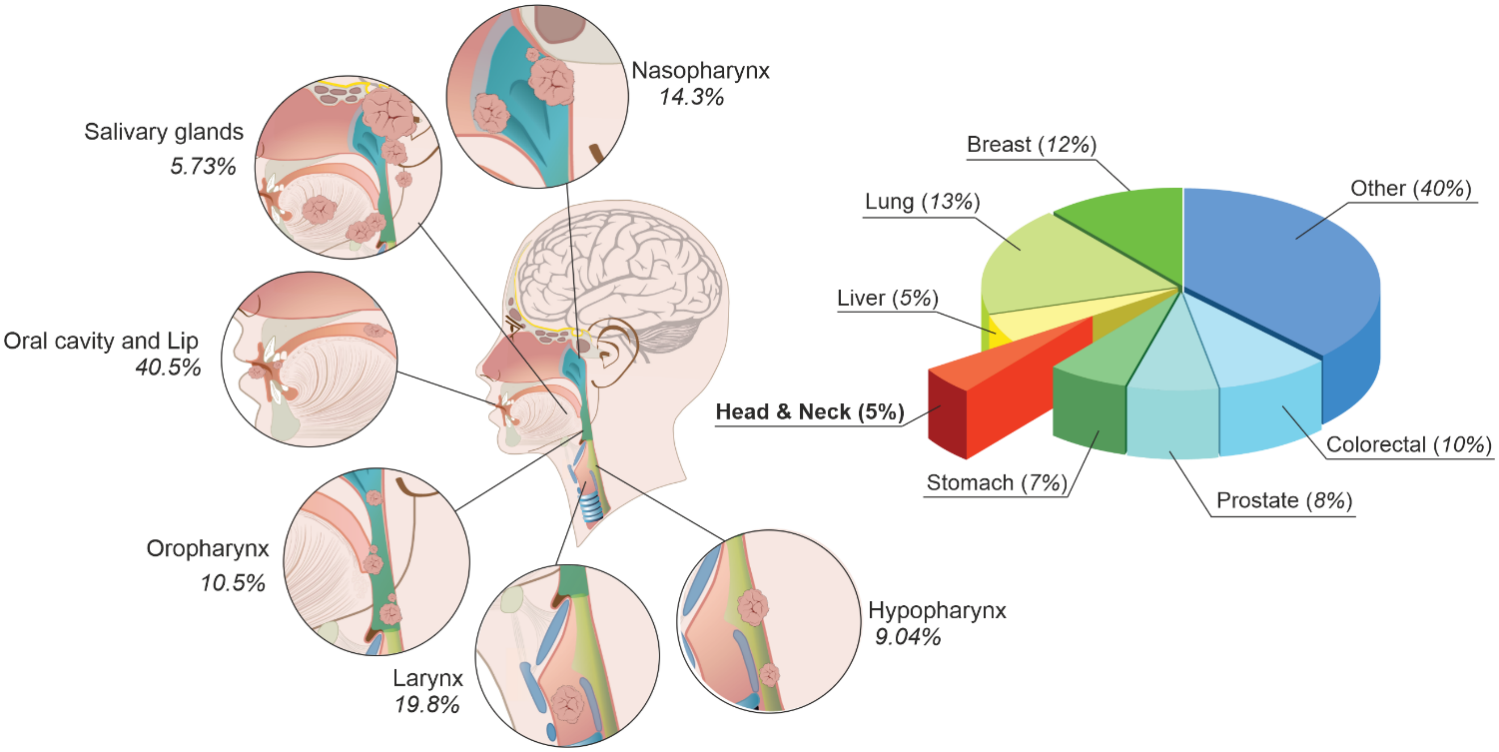
Cancer statistics and head and neck cancer classification.

As can be seen in **Figure 1**, H&N cancer accounts for 5% of all cancer cases worldwide (6). Although H&N cancer accounts for a smaller percentage compared to other cancers, its incidence rate continues to rise. It is estimated that by 2030 the incidence rate will rise by 30% annually (7, 8).

Based on the precise anatomical sites where the cancer arises, head and neck cancer is often divided into several main subregions (**Table 1**). These subregions are important for diagnosing and treating the cancer effectively. Different regions usually have distinct types of squamous cell carcinoma (SCC), i.e., cancer differs in the larynx vs. that in the oral cavity, etc.

**Table 1:** Head and neck cancer subregions and dominant cancer types.

H&N region	Dominant cancer type by HP	Other cancer types in the region
Oral cavity, lip	SCC	BCC, adenocarcinoma
Hypopharynx	SCC	Lymphoma, adenocarcinoma
Larynx	SCC	Chondrosarcoma, melanoma
Nasopharynx	SCC	Adenocarcinoma, lymphoma
Oropharynx	SCC	Melanoma, lymphoma

The most common site for H&N cancer is the oral cavity (including the lip), which accounts for more than 40% of the new cases. The next is laryngeal cancer with almost 20% of all H&N tumors. At third place is nasopharyngeal cancer with almost 15%, followed by hypopharyngeal and oropharyngeal cancer with more than 10% and 9%, respectively. The smallest group of new cancer cases includes salivary gland cancers; they account for more than 5% (6, 9). The most common histopathological (HP) type in this oral and lip region is SCC; it accounts for over 90% of the cases (10). It is followed by rarer HP types, such as basal cell carcinoma (BCC), verrucous carcinoma, adenocarcinoma, adenoid cyst carcinoma, mucoepidermoid carcinoma, lymphoma, sarcoma, and melanoma (11). The majority of larynx malignancies (over 98%) are well differentiated SCC (12). The other types that can be found in HP assessment could be chondrosarcomas, leiomyosarcomas, and melanomas (13). The vast majority of oropharyngeal cancer cases are SCC (over 95%) (14). The other HP types that can be found in the oropharynx are melanoma, primary lymphoid malignant tumors, minor salivary gland tumors, sarcomas, etc. (15). The dominant HP type (over 95%) in the hypopharynx is SCC (16). Other tumor types include lymphoma, sarcoma, and adenocarcinoma (17). In other subsites, SCC carcinoma is found as well in the vast majority of nasopharyngeal cases (>95%) (18). The less frequent types of HP are adenocarcinoma and lymphoma (19). While SCC stands as the most prevalent cancer type, its behavior differs based on the site as well as the influencing developmental factors.

Despite advances in detection and therapy, more than 65% of people with head and neck squamous cell carcinoma have recurrent or metastatic disease (or both) (20). Distant metastases to the head and neck are uncommon compared to other regions, but in 20%–35% of instances, they may be the first sign of an otherwise undetected malignancy (21). Additionally, distant metastases significantly impact the prognosis, often signaling a widespread or disseminated disease. The standard treatment for the head and neck region depends on the site, often encompassing a surgical intervention if viable, along with radiotherapy, chemotherapy, or a combination of these methods. Despite a complex, site-specific treatment, a large number of patients will experience disease recurrence, with up to 60% of patients experiencing local failure and 30% experiencing distant failure (22). There is a high risk to develop a second primary tumor after remission. The risk is up to 2.18% in the follow-up period of 31 months and drops to 1.59% for a period of 10 years but does not decrease further in a period of 30 years (23).

**Figure 2:**
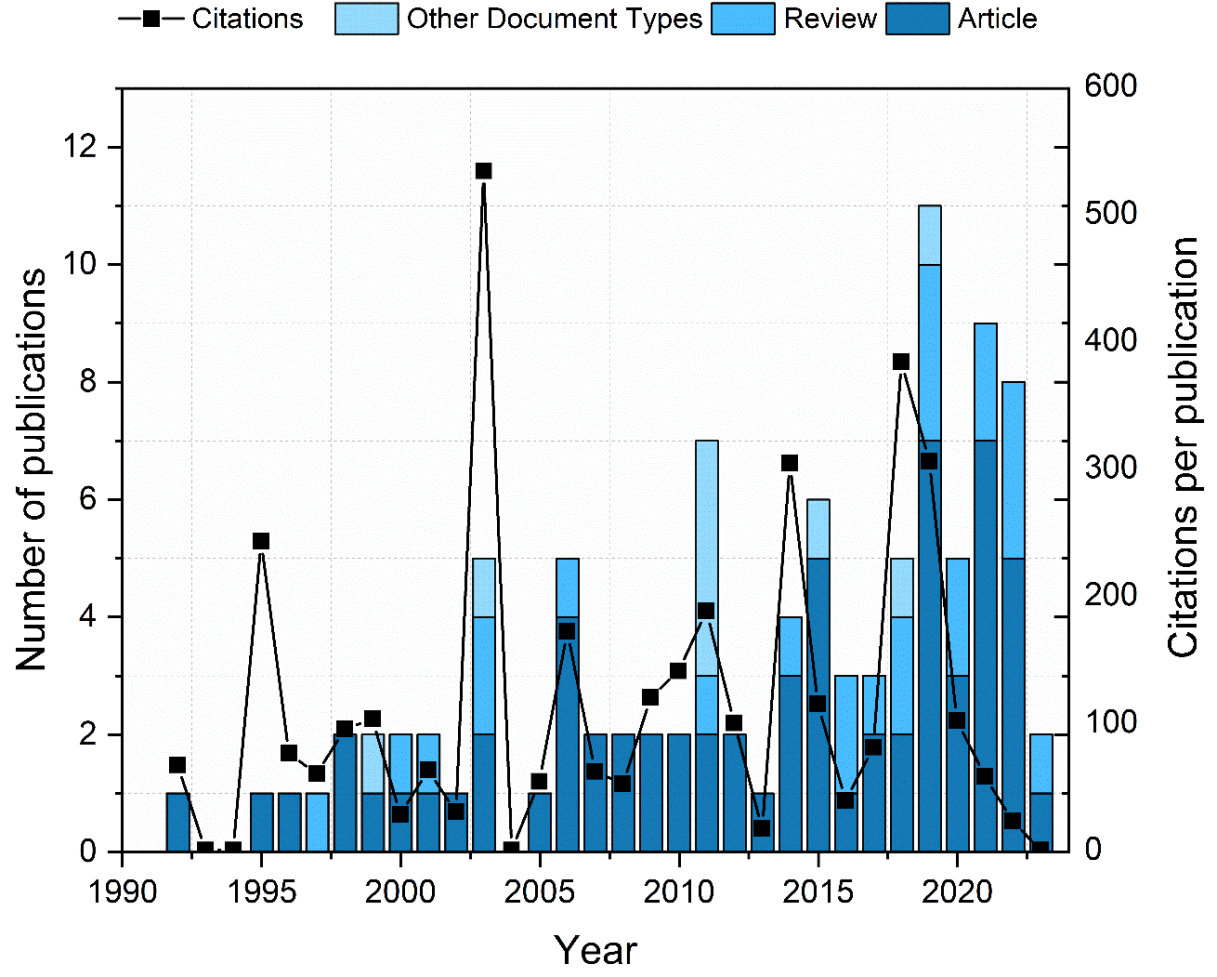
Number of publications and citations in electroporation head and neck cancer research area. Different colors represent the publication types.

The treatment of recurrences is extremely challenging for physicians. In many instances, radiotherapy is no longer a viable choice due to prior irradiation, potential toxicity, and apprehensions about the impacts on function and quality of life (24). For the patients, when all treatment options are exhausted, they are left with palliative treatment. This option usually involves chemotherapy. It might involve single-agent chemotherapy, combinations of platinum-based doublets, or even triplet drug options (25). However, this therapy typically fails to improve the overall survival rate and can reduce the quality of life (26). Due to these reasons, novel treatment methods are on high demand. Among these approaches is electrochemotherapy (ECT), which displays encouraging outcomes by demonstrating high effectiveness and minimal side effects while preserving organ function. Nevertheless, the number of publications (Clarivate Analytics Web of Science, CA WoS) that specifically addresses ECT in head and neck cancer is still low (**Figure 2**), while the application of ECT in the context of solid organs is by several orders of magnitude higher (27). Based on CA WoS, there are currently only 98 papers featuring the keywords “electroporation” and “head and neck cancer” (access date: 2023-11-06).

This paper has reviewed the available literature, characterized the potential applications of ECT in H&N cancer context, systemized available clinical cases, and identified the limitations or challenges, which might affect the success rate of electroporation-based treatment for H&N cancer treatment.

## 2 Electrochemotherapy and its application

An effective and viable alternative to traditional anticancer procedures lies in the utilization of electroporation-based treatment known as electrochemotherapy. ECT can serve as a valuable complement to enhance treatment outcomes while simultaneously reducing the side effects associated with conventional therapies (28). This approach ensures a safe and highly efficient procedure (75% to 99% tumor response) when employed in the treatment of subcutaneous and cutaneous lesions as well as metastases originating from various tumor types (28). ECT is a localized therapeutic approach, which combines the administration of electric pulses capable of temporary permeabilizing cell membrane and thus enables the intracellular delivery of chemotherapeutic drugs like bleomycin (BLM) or cisplatin (CP) (29). Adequate treatment planning ensures no harm to healthy cells or critical blood vessels in close proximity with the tumors (28), while the doses of drugs that are utilized by ECT are below minimal inhibitory concentrations if used without PEF, which minimizes the side effects and improves the tolerance of the procedure by patients. As a result, ECT can be effectively utilized in cases when the tumors are unresectable (30).

**Figure 3:**
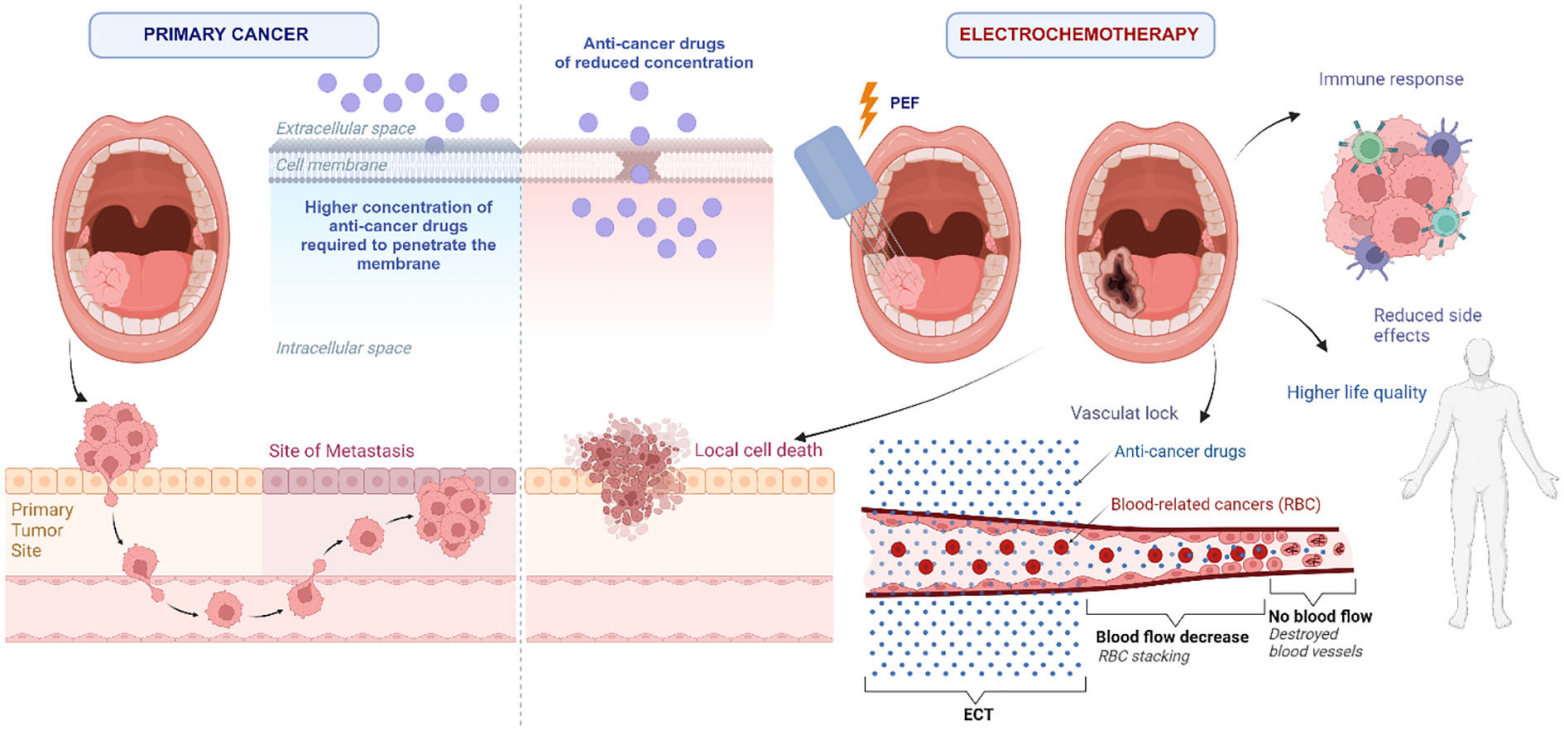
Expected effects of electrochemotherapy in the context of oral cancer.

Additionally, ECT induces the release of damage-associated pattern molecules after treatment, leading to the increase of tumor antigens, which improves the immune reaction against tumor and increases the drug’s efficacy (31, 32). Immune checkpoint inhibitory therapy’s synergistic effects can further stimulate the antitumor immune response. Another phenomenon observed during ECT is the “vascular lock,” which specifically involves vascular disruption and hypoperfusion. The application of ECT creates a decreased blood flow in treated areas after the procedure. This increases the time of drug presence in the targeted volume and could prevent bleeding from well-vascularized structures (31). The principles of electrochemotherapy in the context of oral cancer are summarized in **Figure 3**.

The illustration of primary cancer refers to cancer that is not treated with electrochemotherapy and so requires a larger concentration of anti-cancer drugs to penetrate the cell membrane. While a larger concentration can be used, it may have a detrimental effect on healthy cells. Using a lower dose, on the other hand, increases the risk of primary tumor metastasis. At the same time, ECT provides localized treatment for cancerous tissues by utilizing reduced concentrations of anti-cancer drugs, thus avoiding damage to healthy tissues in the vicinity and thereby reducing the side effects and improving the quality of life for patients. Additionally, as mentioned above, ECT elicits an immune response and, due to destroyed blood vessels, helps prevent bleeding of the lesion following the treatment.

Currently, BLM, and cisplatin (CDDP) are the two drugs routinely used to treat cancer in the context of ECT (33). Bleomycin is a water-soluble glycopeptide antibiotic that breaks DNA, creating DNA fragmentation, chromosomal gaps, and deletions. So far, it has been employed in electroporation-based treatments of different cancers, including H&N (34). Cisplatin is a chemotherapeutic agent, which contains platinum. When cisplatin binds to DNA, it creates intrastrand and interstrand cross-links that cause DNA to adduct and prevent DNA replication and transcription and eventually cause cell death (35). When cisplatin enters the cell, it goes through a series of biochemical processes that produce its active metabolites, which can bind to DNA and crosslink it. Electroporation enhances cisplatin absorption into the cancer cells by increasing the DNA adduct production and boosting the cytotoxicity (36).

Other ECT agents, such as doxorubicin, 5-fluorouracil, gemcitabine, mitomycin C, and calcium, are also being introduced to the field (37). As an alternative to conventional ECT, the most promising so far is calcium ECT (38). The approach entails the precise intracellular delivery of cytotoxic calcium concentrations, causing ATP depletion, which results in cancer cell death and tumor necrosis (39). Additionally, elevated intracellular calcium levels open up mitochondrial pores, resulting in the dissipation of electrochemical gradient and formation of new ATPases (40). The other effect related to calcium and cell death is that it activates proteases and lipases and generates reactive oxygen species, which can also contribute to apoptosis (40, 41). Since calcium electroporation does not fall under the category of antineoplastic drugs, it is exempt from the precautions associated with these drugs with regard to storage, handling, disposal, toxicity, and mutagenicity. Calcium electroporation effectively induces tumor necrosis while causing less damage to normal tissue, making it a very promising ion for further clinical evaluation (42). It was already tested in clinical studies (43), including H&N cancer (44). Calcium is typically administered intratumorally, while BLM and CDDP are delivered either intratumorally or intravenously (37, 44).

## 3 Chemo/radio-resistance and electroporation

**Figure 4:**
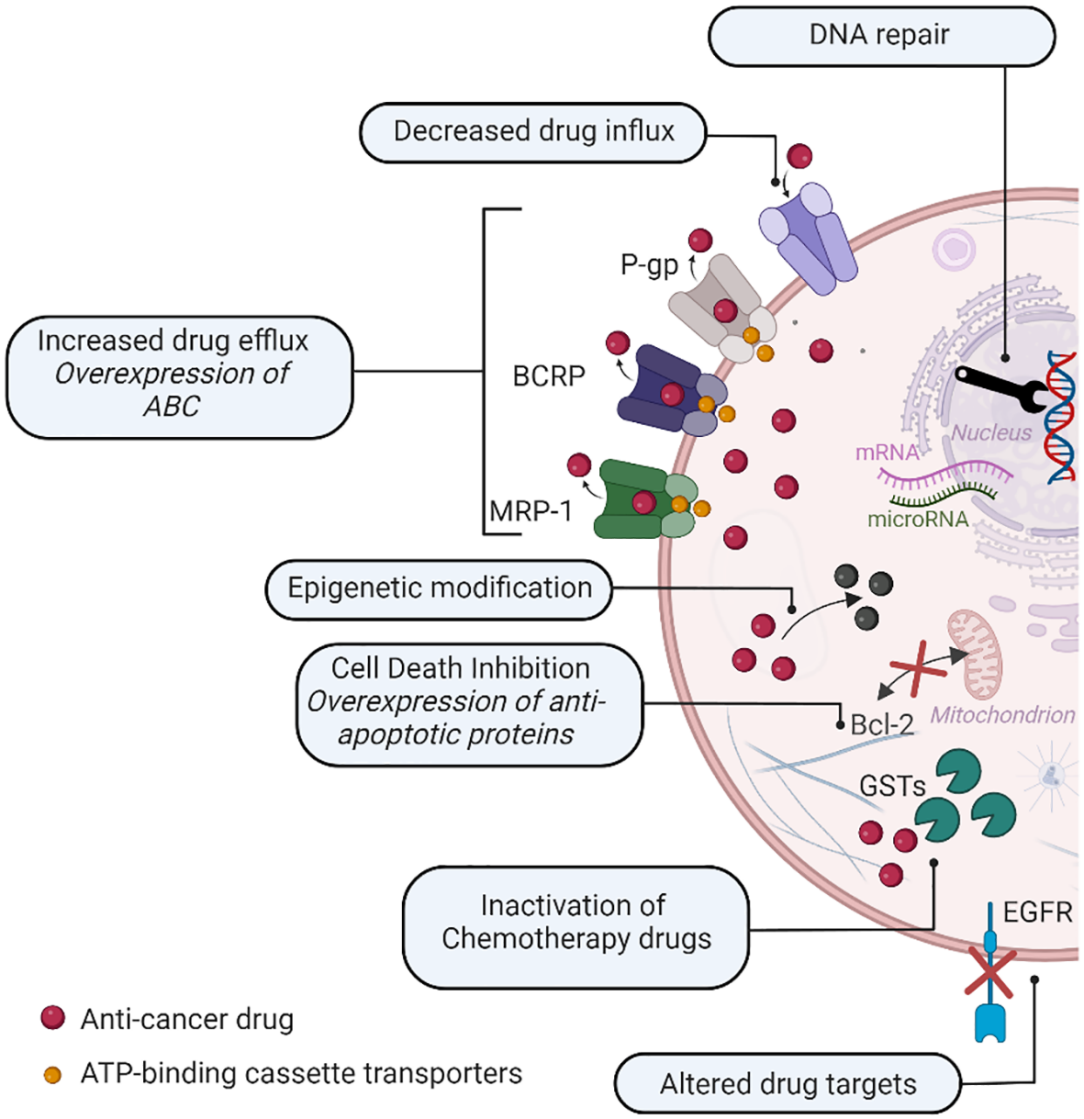
Multi-drug resistance causing mechanisms in cancer cells.

Although many cancers respond to chemotherapy, a major obstacle to the treatment is multiple drug resistance (MDR). Based on the factors involved, drug resistance can be split into two categories: intrinsic resistance and extrinsic resistance (45). Intrinsic resistance implies the elements that are present in cancer cells or tissues themselves and responsible for lowering the efficiency of cancer chemotherapeutics prior to receiving chemotherapy. Extrinsic or acquired drug resistance, on the other hand, can arise during the treatment of tumors that were initially responsive to cytotoxic drugs and counteract their therapeutic effects as a result of a variety of adaptive responses, such as an increase in the expression of the therapeutic target and activation of alternative signaling pathways (45, 46). There are a lot of factors involved in MDR, and the main mechanisms of drug resistance are shown in **Figure 4**.

As can be seen in **Figure 4**, the main causes for MDR are as follows:

• Increased drug efflux: overexpression of ATP-binding cassette transporters (ABC), such as BCRL, MRP1, or P-glycoprotein (P-gp), which is one of the main causes of drug resistance. The intracellular concentration and effectiveness of chemotherapeutic medications are decreased by ABC transporting chemotherapy agents out of cancer cells (47, 48).
• Altered drug targets: Cancer cells may acquire genetic alterations that modify the targets of chemotherapy treatments, rendering them ineffective—for instance, mutations in drug target genes like the epidermal growth factor receptor (EGFR) in lung cancer, leading to resistance to EGFR inhibitors (49, 50).
• Inactivation of chemotherapy drugs: Cancer cells might produce enzymes capable of metabolizing chemotherapy agents and turning them into inactive variants. Resistance to platinum-based medicines, such as cisplatin, is characterized by an increased production of enzymes such as glutathione S-transferases (GSTs), which neutralizes the drug (48, 51).
• DNA repair mechanisms: In cancer cells, chemotherapy medicines cause DNA damage. However, enhanced DNA repair capacity can provide resistance by efficiently repairing drug-induced DNA damages *via* the activation of repair pathways such as nucleotide excision repair and homologous recombination (52, 53).
• Cell death inhibition: Cancer cells can develop a variety of ways to avoid chemotherapy-induced cell death, allowing them to survive and proliferate. Changes in apoptotic pathways (e.g., Bcl-2 protein family mutations), such as the overexpression of anti-apoptotic proteins, can prevent apoptosis and provide resistance to chemotherapy (54, 55). Furthermore, abnormalities in cell death signaling networks, such as the activation of survival pathways, might enhance cell survival and mitigate the cytotoxic effects of chemotherapy medications (56).
• Epigenetic modification: Epigenetic changes, such as DNA methylation and histone modifications, can affect gene expression patterns, contributing to drug resistance. These changes can quiet tumor suppressor genes or activate drug resistance genes, boosting cell survival and decreasing treatment sensitivity (57, 58).

The same issue arises not only with chemotherapy but also with radiotherapy. Radiotherapy resistance (RR) can occur due to multiple factors, including genetic mutations, altered DNA repair mechanisms, and changes in the tumor microenvironment (59). Among the various mechanisms proposed as potential reasons of RR, hypoxia has been emphasized. The presence of oxygen in the tissues enhances the effects of ionizing radiation; tumors with lower oxygen levels tend to exhibit greater resistance to ionizing radiation damage (60). Hypoxic regions are common in H&N SCC and may be related with resistance to ionizing radiation (61). Changes in various intracellular pathways, especially those involved in DNA repair, cell replication, cell cycle, and apoptosis, have been found to counteract ionizing radiation-induced cell death and so result in RR (62). There are numerous routes involved in H&N cancer RR, and it is one of the reasons why it is so difficult to overcome this obstacle. There is a need for new radio-sensitizing agents, but unfortunately most of the clinical trials fail during evaluation (63). There is a great need for alternative therapeutic modalities in the context of H&N cancer that will be able to overcome chemo/radio-resistance.

Electrochemotherapy is indicated as one of the viable options to control cancer MDR. Since ECT triggers the increased cell chemotherapeutic agent’s uptake, cancerous cells become less capable of maintaining their intracellular parts and suppress DNA repair capabilities (27). M. Condello et al. demonstrated that *in vitro* EP with mitomycin C increased the drug cytotoxicity in oral and laryngeal cancer cells with intrinsic drug resistance compared to a single treatment by inhibiting the protective mechanism of autophagy in favor of apoptosis (64). Cemazar et al. concluded that membrane restriction is the major obstacle to cisplatin cytotoxicity in cisplatin-resistant murine sarcoma cells. Their study showed that cells were equally sensitive to cisplatin ECT *in vitro*. Similarly, the *in vivo* results indicated an increased effectiveness of cisplatin in both parental and cisplatin-resistant tumors, though a significantly higher cure rate was assessed in cisplatin-sensitive compared to cisplatin-resistant tumors: 85% and 6%, respectively, at the same 8-mg/kg dose (65). Thus, MDR remains a challenge even in electroporation-based treatments. Recently, novel methods and hypothesis over MDR-related cell response in the context of ECT were proposed. The utilization of multi-drug cocktails, namely, the combinations of different anti-cancer agents, is also possible (66). Such approach decreases the probability of drug resistance and minimizes concurrent toxicity while effectively eliminating tumor cells. MDR may also be managed through modulation of PEF parameters. Shorter-duration electric pulses (nanosecond range) were shown to be more effective in countering drug resistance mechanisms compared to conventional European Standard Operating Procedures of Electrochemotherapy (ESOPE) protocols employing 100-µs pulse trains (66).

## 4 Conventional treatments *versus* ECT

For locally or locoregionally limited head and neck squamous cell carcinoma (HNSCC), surgery, radiotherapy (RT), and chemotherapy (CT) are the main treatment techniques. Planning a course of treatment should maximize function preservation while aiming for the best curative strategy. A single-modality intervention can yield cure rates of more than 80% for individuals with a small primary malignancy that involves just one node or with no clinical nodal involvement (resection or radiation) (67). Surgical resection followed by adjuvant RT or concurrent chemoradiation (CRT) with salvage surgery acting as salvage therapy—also known as the organ preservation approach—remains the cornerstone of treatment for locoregionally progressed HNSCC (68). However, every case is unique, and treatment is tailored to each patient, e.g., in the oral cavity, stage I and II cancers are usually treated with primary surgery or RT, while stage III and IV cases are treated with surgery followed by RT with or without CRT (69). The treatment method is dependent on the anatomical site, cancer type, stage of the disease, and general health of the patient.

Besides the benefits, most treatments have their own limitations and disadvantages. One major concern with CT is the significant side effects including nausea, fatigue, hair loss, neutropenia, and general toxicity (70). When comparing the side effects, cisplatin-based chemotherapy yields more adverse effects (toxicity, mucositis, nausea, vomiting), when compared to RT alone (71). Because ECT requires a smaller drug dose compared to CT, it leads to less toxicity and a reduced rate of side effects (72). Moreover, chemotherapy is a non-specific treatment, affecting both cancerous and healthy dividing cells. This lack of specificity leads to collateral damage to healthy tissues. This indiscriminate action can result in persistent issues such as compromised immune function, cardiovascular problems, and impaired cognitive abilities, affecting the overall health and wellbeing of individuals who have undergone chemotherapy (73). When treating HNSCC, it is important to consider both aesthetics and function. The impact of surgical therapy is primarily determined by site. The function is mostly compromised while surgically treating sites like tongue, lip, or larynx. When treating cancer on the face or nose, aesthetics is also important. ECT is effective in both of these circumstances since the function is nearly not impacted and the visual element is far less impaired than with surgery; it requires as well only one or two treatments to achieve high OR (74). Both CT and RT can lead to esthetic changes, mostly on pigmentation of the skin (75, 76). Side effects, like impaired function, depend on the treated site, and it can vary from difficulty in swallowing, xerostomia, and others (77). CT can lead to long-term sequelae, presenting challenges that extend beyond the active treatment period. One notable consequence is the potential for lasting damage to healthy cells and tissues. Less toxic treatment approaches are needed since chemotherapy-induced toxicity seems to be a major problem in the treatment of HNSCC (78). Furthermore, CT has been associated with an increased risk of secondary cancers. The exposure to these agents during the initial treatment may contribute to the development of new malignancies later in life, underscoring the complex and multifaceted nature of chemotherapy-related sequelae (79). The same goes for radiotherapy; in some cases, it increases secondary cancer risk in different locations (80, 81). This is because both CT and radiotherapy not only impact cancerous cells and cancer itself but also affect all the body cells, as in the case of CT, or specific areas and cells in the case of RT. There is also data that some chemotherapeutic drugs and radiotherapy are linked to a higher incidence of cardiovascular diseases (82). The various aspects of cancer treatments are summarized in **Table 2**.

**Table 2:** Summary of typical cancer treatment methods.

Aspect	Radiotherapy	Chemotherapy	Surgery	ECT
Targeted area	Limited to the treated region, with minimal damage to surrounding healthy tissues	Systemic, affecting the entire body and impacting both cancerous and healthy cells	Localized, focusing on the removal of the tumor and surrounding tissues	Localized, based on electrodes and electric field spatial distribution
Side effects	Depends on the treated site and area, including fatigue, hair loss, nausea, vomiting; increased secondary cancer risk	Widespread side effects, including nausea, vomiting, fatigue, hair loss, general long-term toxicity; increased secondary cancer risk	Varies based on the extent of the surgery, including pain, potential scarring, and impaired function	Bleeding, ulceration of treated area, muscle contraction during pulse application; mild to moderate pain depending on the treatment site
Treatment duration	Multiple sessions during the time period	Administered in cycles, with treatment periods and breaks to allow recovery	Duration varies based on the complexity of the surgery and recovery time	Usually one or two procedures
Esthetics and function of the targeted area	Depends on the site, including skin changes, impaired function of the site	Minimal impact on function and esthetics	Depends on the scope of surgery, could lead to impaired function and esthetics	Minimal impact on esthetics and function of treated area
Limitations	Radio-resistance, maximum dosage	Multidrug resistance, maximum dosage of the drug	Success/possibility of surgery depends on the extent surrounding neurovascular structures	The treatment success depends on spatial pulsed electric field distribution, which is highly affected by tissue heterogeneity, requires direct contact of the electrodes with the tumor

## 5 H&N tumors in clinical ECT studies

The response of individual tumors to ECT in clinical setting was classified by Mali et al. as complete response (CR), partial response (PR), no change (NC), or progressive disease (PD) (32). The concepts of objective response (OR, including CR and PR) and no response were introduced (32). This evaluation method is still applied today as Response Evaluation Criteria in Solid Tumors (RECIST). It is widely used to evaluate tumor response to ECT. In the first ECT studies, different protocols for electrochemotherapy were performed with different doses of chemotherapy drugs, different pulse parameters, and different electric pulse generators in conjunction with different electrode types. The first standard operating procedure (SOP) was introduced in 2006 as ESOPE (83). It standardized inclusion criteria, drug doses, pulse parameters, patient follow-up, and more. The main disadvantage is that it focuses mainly on skin cancers and metastases. It was updated in 2018 with new recommendations for indications for electrochemotherapy, pretreatment information and evaluation, and treatment choices as well as follow-up (84).

Most of the studies concentrate on palliative treatment objectives, primarily relying on the current standard operating procedure (SOP) of ESOPE. The prevailing inclusion criteria are outlined as follows:

— Cutaneous metastases, of any histology, which are symptomatic due to bleeding, ulceration, oozing, Odorkor pain.
— Progression of cutaneous metastases, where the development of symptoms, as listed above, is expected.
— Primary skin cancers, including recurrent tumors, where other treatment modalities (surgery, radiotherapy, and systemic therapies) have failed or are not possible.
— Patients who are receiving systemic therapy, but where cutaneous metastases are progressing or not responding despite a satisfactory response to systemic therapy in internal organs.
— Patient preference for electrochemotherapy after other treatment possibilities have been thoroughly explained to the patient (84).

All the studies and protocols involved and mentioned going forward are summarized and presented in **Supplementary Table S3**.

The DAHANCA 32, a clinical phase 2 study published in 2018, demonstrated promising results of recurrent mucosal head and neck tumor treatment using ECT. All 26 patients included in the study were pretreated with radiotherapy and had no other curative options. The treatment was conducted according to ESOPE guidelines with intravenous bleomycin injection (15,000 IU/m^2^). The overall tumor response rate was 58% by CT RESIST criteria (five tumors demonstrated a complete response and 10 had partial). Moreover, 16 out of 17 performed post-treatment biopsies (4 weeks after ECT) showed no remaining cancer cells (85). An analogous previous multicentral study of 43 cases, the EURECA project (2017), showed an overall response of 56%. The only factor influencing outcomes was tumor size, and the response rates were higher if the tumor was smaller than 3 cm (86).

A 2018 prospective study by B. Pichi et al., including 36 cases of any type of recurrent head and neck tumors, demonstrated a 100% overall response rate, though it was mostly partial (72). A similar study of 93 cases conducted by F. Longo et al. in 2019 showed 45% overall response with 5% complete response as well as improved pain and bleeding control (87). In both studies, the median survival rate was 9 months (72, 87).

A study by G. Riva et al. demonstrated a significantly higher global health status and social functioning in patients treated with ECT for head and neck tumors as well as decreased use of pain medications and better appetite (88). The previously mentioned EURECA project demonstrated an increase in wellbeing at 2 and 4 months with the EQ-5D questionnaire, unchanged scores with the EORTC QLQ-C30 (except “diarrhea”), and the EORTC QLQ-H&N35 (except significantly poorer swallowing) questionnaires (86). The DAHANCA study showed similar results (85).

One of the largest recent studies was conducted by Longo et al. in 2019 (87). In this setting, all 93 patients had a recurrent and/or metastatic disease and were treated with at least two chemotherapy and/or radiotherapy lines and were not suitable for surgery or chemo/radiotherapy. The overall response (OR) rate was 45%, with a complete response rate (CR) of 5%. This research found that the OR rate was significantly influenced by the tumor size. If the tumor was smaller than 3 cm (30 patients), the OR rate was 54.5%; if it was larger than 3 cm (10 patients), the OR rate was 26.3%. Similar findings were found in other studies that measured the size of the tumor. Most of the studies showed that smaller tumors (≤3 cm) have better response to ECT compared to larger tumors (88–91).

The tumor size and treatment response correlation was even bigger in the study done by Gargulio et al. (92). The treatment response based on tumor size was 89.5% CR for lesions of 4 cm or smaller and 16.7% for tumors larger than 4 cm. Similar results were demonstrated by Longo et al. (87). This study found that smaller tumors respond better to ECT (>3 cm OR rate 88%, <3 m OR rate 68%) without depending on tumor histology. The negative relationship between tumor response and larger size can be explained both technically (inadequate covering of the cancer by electric fields) and pharmacologically (irregular drug distribution inside the tumor). Another important finding regarding tumor response to the treatment was done by Claussen et al. in 2022. They evaluated tumor response in relation to its ulceration. It proved that non-ulcerated lesions respond to ECT significantly better than ulcerated lesions. This study enrolled a large number of patients (716). The non-ulcerated group had OR of 86% and CR of 65% compared to the ulcerated group’s OR of 79% and CR of 51% (93). It is important to mention that the median lesion size in the non-ulcerated group was twice smaller than in the ulcerated group (15 and 30 mm, respectively), and previous studies demonstrated that smaller tumors respond better to ECT. The relationship between ulceration and tumor response rate may be explained by local hypoxic metabolism or insufficient angiogenesis, resulting in an increase in toxic products (94, 95).

Not all studies were conducted on a palliative treatment basis. A 2015 paper by Landström et al. (96) focused on curative intent for patients with SCC, and 19 patients were included in the study. Patients with tumors deeper than 5 mm received radiotherapy in addition to ECT. The 5-year survival rate was 63.2% (12/19), and there was no local recurrence observed in patients who died during follow-up. It is important to mention that, in this study, they used higher bleomycin doses than in the ESOPE protocol (1,000 IU/cm^3^, ESOPE 250 IU/cm^3^). In addition, the calculation of the treatment volume differed from the ESOPE protocol (addition of 1-cm margins compared to ESOPE protocol). This means that high survivability could be linked to higher doses of bleomycin injected intratumorally.

A similar study was done by Bertino et al. in 2016 (89). This study focused on evaluating the efficacy of ECT in local tumor control as a primary treatment (89). They found that primary tumors respond better to ECT than recurrent or metastatic tumors. For the primary tumors (50 patients), CR was 70%, PR 20%, SD 8%, and PD 8%. While for secondary tumors (49 patients), CR was 55%, PR 18.5%, SD 18.5%, and PD 6%. This shows that untreated tumors are more responsive to ECT, and it may be considered as a first-line treatment in some cases. It is imperative to note that recurrent tumors are usually large and irregularly shaped, with deep margins buried under the apparent surface (97). Therefore, covering the whole tumor with a suitable electric field cannot be guaranteed in every situation. Additionally, scar tissue and impaired vascularization from previous treatments may result in reduced bleomycin delivery to tumor cells during electroporation (98).

A study done by Campana et al. in 2014 (99) used cisplatin for treating H&N cancer. It was given intratumorally. It was a small group of two patients, and both were refractory to treatment. In this case, cisplatin showed unsatisfactory results, but previously conducted studies were promising for using cisplatin in ECT (100).

In a 2019 study that was conducted by Jamsek et al. with reduced doses of bleomycin (101), 28 patients, 65 years or older, were treated. The control group consisted of 16 patients who were administrated standard doses of bleomycin (15.000 IU/m^2^ body surface area). Meanwhile, the experimental group included 12 patients; they received reduced bleomycin dosage (10,000 IU/m^2^ body surface area). No statistically significant differences were found between the two groups with respect to patient (age, gender) and tumor (diameter, histology, recurrent disease) characteristics (101). This implies that drug dosage, along with its associated toxicity and undesirable effects, could be lessened, potentially resulting in reduced overall toxicity and adverse effects. The study also showed that there was no difference between skin tumors of the head and neck and SCC of the oral cavity or oropharynx or between different electrodes or histological types of tumors, although basal cell carcinoma (BCC) responded better than other types of cancer.

This is evident in a recent comprehensive study involving a large patient cohort (330 patients, 623 tumors) done by Bertino G et al. in 2022 (102) (data was taken from InsPECT database), where OR per tumor was of 93% and CR was 83.1%, though the findings vary when compared to the work of the same author, which concentrated on cutaneous SCC (103). This study compared the data of 162 patients with 342 tumors. OR was 79% and CR was 61%. BCC OR and CR are significantly higher than SCC. This could be due the nature of tumor or tumor extent and, most notably, tumor histology.

Another study focusing on a completely different tumor histology—angiosarcomas—conducted in 2016 by Guida et al. (104) based its design on ESOPE. Its results did not significantly differ from those of other studies predominantly featuring SCC. The tumor response rates were as follows: OR: 85.5%, CR: 66.7%, PR: 18.5%, SD: 9.3%, and PD: 5.6% of the total number of tumors.

Plaschke et al., in 2017, showed that patients who were pretreated with chemo/radiotherapy and or surgery resulted in 56% of OR and 19% of CR. Similar findings were observed in the study done by Di Monta in 2017, in which CR was only 22.7% and OR was 81%. The conclusion drawn was that those studies achieving higher complete response rates are associated with lower tumor stages and a patient cohort displaying less malignant tumor tendencies (105). Meanwhile, other studies demonstrated better results—for instance, in studies conducted by Gargiulio et al. in 2012 and 2018, the tumor responses demonstrated higher rates: achieving an overall response (OR) rate of 100%, a complete response (CR) rate of 72% (92), and an OR rate of 100% with a CR of 71.4% (106), respectively. The cohort group for this research was patients with SCC of the lower lip. Higher response rates were achieved in T1–T2 tumors, and it served as reliable neo-adjuvant therapy for patients with T3 tumors (106).

The most successful study in terms of OR and CR was conducted by Landström et al. in 2015. Both OR and CR were 100% (96). All patients had mucosal primary tumor T1 or T2 (oral cavity or oropharynx). Among the 12 surviving patients, the 5-year local control maintained a rate of 100%, while the 5-year tumor-specific survival stood at 75%. It is important to highlight that 12 out of 19 patients underwent radiotherapy (RT) subsequent to receiving ECT. Additionally, for two patients, neck dissection (ND) was performed concurrently with ECT, and for four patients, it was carried out subsequently. Consequently, this situation cannot be solely attributed to true ECT survivability.

When comparing the treatment response results, we get quite different numbers of OR. Pinpointing an exact reason is challenging due to the inherent heterogeneity within the patient population. Moreover, most of the patients in the studies are pretreated, or all treatment options have been exhausted (72, 89, 90, 107). While the overall response rate might not be particularly high, considering that these are patients who have exhausted all treatment options, it could potentially enhance the overall survival and quality of life—for instance, in a study done by Riva in 2021, all patients were without any other curative treatment options. In this case, OR was 48% (11% CR, 37% PR) (88).

It should be highlighted that, according to current clinical practice, ECT is typically applied for older patients when other treatment options are unsuitable due to age and health risks. To evaluate the efficiency of ECT, the research distinguished two groups: patients older than 90 years old and those younger than 90 years old. The local response of the first group (>90 years old) was similar to that of the group composed of younger patients (<90 years old): OR: 87%, CR: 57% and OR: 88%, CR: 65%, respectively. These findings confirm ECT as a viable treatment choice independent of patient’s age; however, it is indicatory that the response rates could be even better if ECT was used as a primary option for tumor treatment rather than as a last resort.

## 6 Electrochemotherapy-related toxicity and quality of life

Currently, ECT is frequently used with a palliative intent, while adverse effects, toxicity, and changes in quality of life are also very important. A 2021 systemic review of ECT in mucosal head and neck cancers states that the majority of patients in the included studies had no serious adverse events during and immediately after ECT. At 1 or 2 days later, swelling of the treated site appears, which is followed by necrotic and healing phases. Therefore, a tracheostomy may be needed to avoid upper airway obstruction in some cases. No changes or significant reduction of pain scores and significant improvement in bleeding control were seen in month 1 or 2 post-ECT. No bleomycin toxicity was reported (108).

Nonetheless, the most relevant side effects of ECT-treated H&N cancer were listed by Campana et al. (99). Their research outlined that the effects of electrochemotherapy can cause mild tissue damage or complications, which are not frequent though, local toxicity and tissue swelling, soft tissue necrosis, soft tissue or bone infection, bleeding, pain associated with wound healing, etc.

Quaglino et al. performed a pain assessment before the treatment as well as within 24 h, on day 45, and more than 45 days post-treatment using numeric rating scale (NRS) (109). In total, 121 patients with metastatic melanoma, squamous cell carcinoma, breast cancer, basal cell carcinoma, and other malignancies developing tumors of 0.3 to 40 cm in sizes were under the scope. The occurrence of post-procedure pain exhibited statistically significant associations with the following factors: the presence of moderate or severe pain before treatment, the size of the tumor, formerly irradiation, and the utilization of high PEF values. Most of the patients (60%) did not feel any pain before ECT. After 24 h, the pain perception in these patients increased; however, it decreased again during the subsequent visit. Other researchers associate unpleased sensations with repetitive ECT treatments (110). Thus, the quantity of the procedures should be limited. These factors can be managed through pre-treatment planning, which involves numerical computations, the selection of the most suitable pulsed electric field (PEF) properties, and the use of an appropriate electrode structure.

## 7 Pulsed electric field parameters and pre-treatment planning of ECT

**Figure 5:**
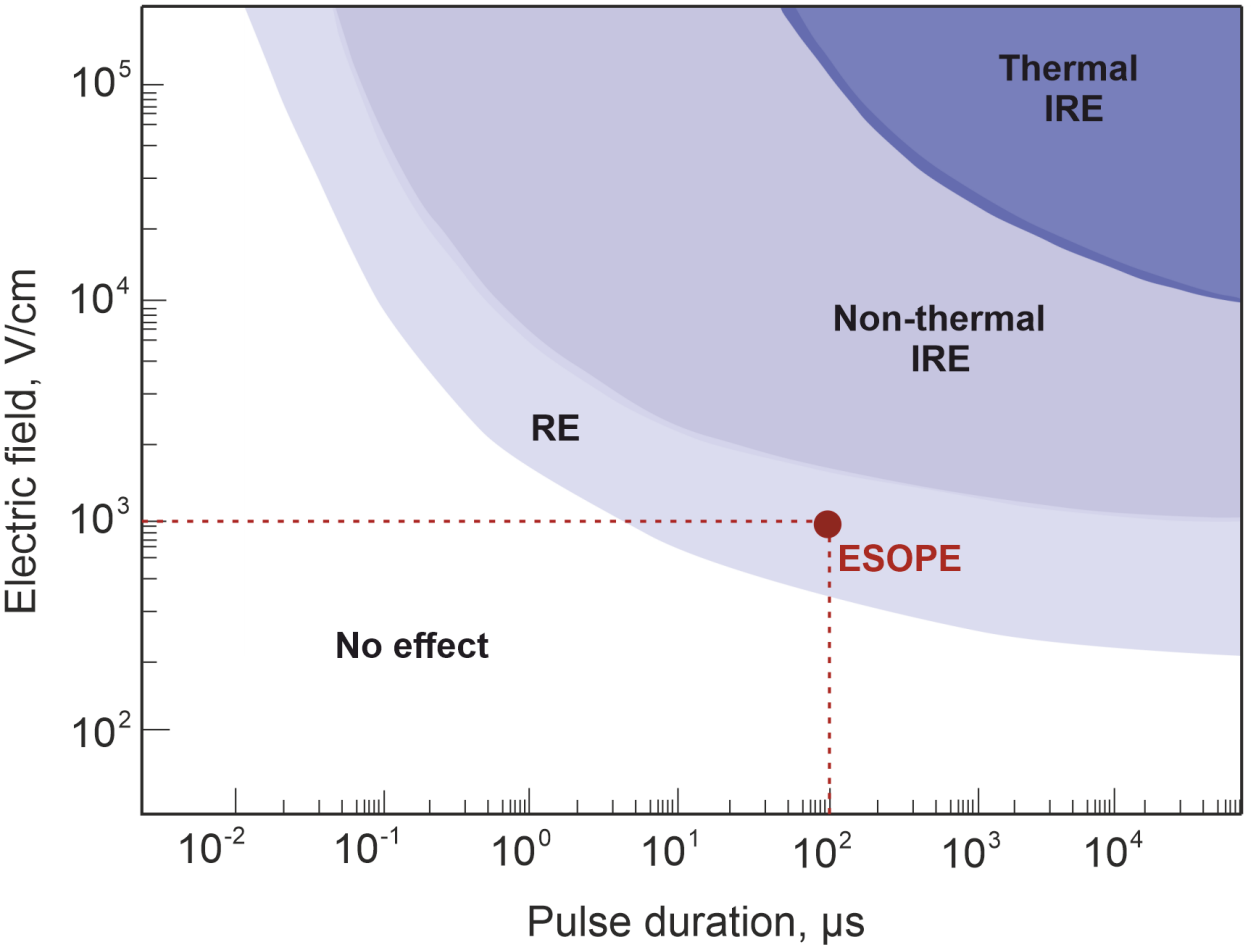
Cell response dependency on pulsed electric field strength and pulse duration. The red circle represents standard European Standard Operating Procedures of Electrochemotherapy treatment protocol (eight 100-µs-duration unipolar pulses with 1 Hz repetition frequency and 800–1,400 V/cm electric field strength (115).

The biological tissues exposed to the electric field of specific properties experience permanent or transient structural changes in their cell level, recognized as irreversible electroporation (IRE) and reversible electroporation (RE), respectively. As mentioned above, the latest mentioned EP method is successfully employed for ECT or gene delivery (GET), allowing the administration of exogenous molecules, such as anti-cancer drugs or DNA, into the cell nucleus (111). Irreversible electroporation, on the contrary, triggers targeted tissue ablation and death of the cancerous cells without administration of external substances (112). Although both methods found the métier of application in clinical cancer treatment setting, ECT is more favorable in H&N cancer (113). Cell response to EP procedure can be manipulated though pulsed electric field parameters: intensity electric field strength and/or pulse duration (114), shown in **Figure 5**, and other parameters such as pulse shape (116), number (117), and pulse repetition frequency (118).

The established properties of PEF as defined by ESOPE are typically adopted in clinical ECT treatment. However, due to minimized muscle contractions, pain, or thermal effects (119), there is growing interest in equivalent PEF properties, especially the potential utilization of pulses with reduced duration (nanosecond), which are proven to minimize neuromuscular stimulation (120) and joule heating (121), simultaneously ensuring efficiency-comparable tumor response. Given this perspective, it is advisable to incorporate these new PEF protocols into clinical settings.

Achieving a favorable treatment outcome is hinged on ensuring that the tumor is sufficiently covered by adequate electric field (122). This becomes a challenge due to the significant heterogeneity of the tissue (123). This implies that morphological difference and dielectric properties of the tissue layers (high resistivity cutaneous or high conductivity due to moisture of the mucous membrane with respect to tumor) may distort the spatial distribution of the electric field within the tissue, leading to an either extensively high or an excessively low PEF value within the tumor (124, 125) and, consequently, causing its partial response or the regrowth of the tumor. Another constituent is the electroporation electrodes, which, depending on contact method, utilization method, and composition, induce PEF within the tissue (126). The electrode design must adhere to safety standards, minimize the invasiveness of the procedure, and prevent bacterial infections (127).

During the clinical procedure, it is not feasible to monitor the spatial electric field distribution in real time; however, the first solutions to use electro-acoustic tomography are being proposed (128). Currently, the optimal approach to predetermine the outcome of individual electroporation-based procedure, including ECT, involves pretreatment planning through numerical models (129), though current treatment planning in SOP does not cover simulation of treatment which would be beneficial.

**Figure 6:**
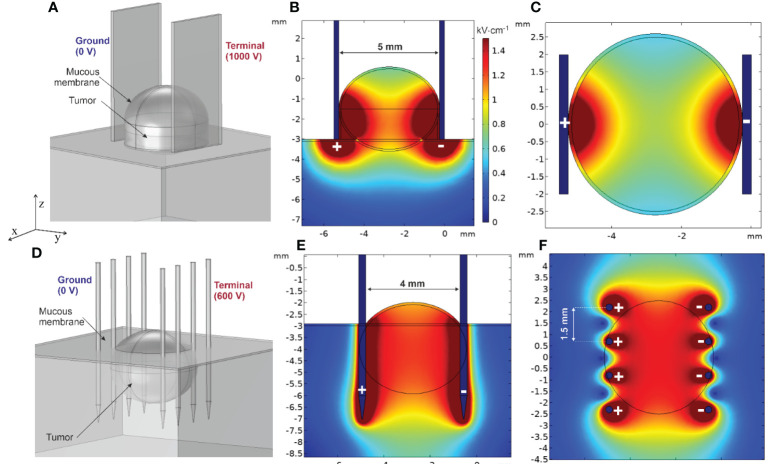
Spatial electric field distribution in the tumor using plate and needle array electrodes: **(A)** tumor finite element model (FEM) using plate electrodes, **(B)** typical spatial electric field distribution using plate electrodes (vertical cross-section, ZY axis), **(C)** typical spatial electric field distribution using plate electrodes (horizontal cross-section, XY axis), **(D)** tumor FEM model using needle array electrodes, **(E)** typical spatial electric field distribution using needle electrodes (vertical cross-section, ZY axis), and **(F)** typical spatial electric field distribution using needle electrodes (horizontal cross-section, XY axis).

Considering previous clinical settings, the simplified tumor model of HNC located in the oral cavity is shown in **Figure 6**. The developed model consists of mass tissue representing the tumor and surrounding tissue covered with a thin layer of mucous membrane (0.1 mm in thickness) with 0.04 and 4.61 S/m conductivities, respectively. Two electrode structures typically employed in ECT for head and neck cancer were tested—non-invasive (plate) and invasive (fixed linear needles), with 5- and 4-mm gap size (**Figures 6A, D**, respectively). Furthermore, 1,000- and 600-V terminal voltages were selected to ensure ~1–1.3 kV/cm PEF inside the tumor depending on the electrode type. When utilizing plate electrodes, ensuring a larger contact area between the electrode and tissue is crucial to adequately envelop the lower part of the tumor with an effective PEF. Forming a lump was deemed beneficial for the studied tumor type and was thus integrated into the finite element model. Conversely, when employing invasive needle array electrodes, compressing the tumor becomes less feasible. To counter this, deeper needle penetration, surpassing the tumor’s depth, was considered for a tumor of equivalent size, as depicted in **Figure 6C**.

Spatial electric field distribution within the tumor and tissues is inhomogeneous, especially in the case of plate electrodes. As per **Figures 6B, C**, the upper and lower segments of the tumor will experience low electric field values. Simultaneously, the surrounding tissue will receive unnecessarily high PEF, though in the context of oral cancers the thin layer of saliva surrounding the lesion slightly improves the field non-homogeneity at the top part of the tumor. Moisture within the oral cavity functions in a manner similar to that of conductive gel used in cutaneous treatments (130). This similarity lies in its ability to facilitate a more consistent distribution of the electric field. However, it is crucial to regulate the salivation (as well as the amount of conductive gel) during the clinical procedure since it could potentially cause short circuit of the electroporator. Needle electrodes, on the contrary, produce a more uniform electric field. The tumor appears to be enveloped by an electric field of approximately ~1.2 kV/cm, suggesting that employing such a method and electrodes could potentially lead to a more successful treatment outcome. Nevertheless, this type of electrode necessitates physical penetration into the tissue, consequently raising concerns about the invasiveness of the procedure and the potential risk of bacterial contamination.

At the same time, skin electroporation is inherently complex, primarily owing to the distinctive structure and low conductivity of the stratum corneum (131). To minimize the invasiveness of the procedure and mitigate the risk of a bacterial infection, it is advisable to employ non-invasive electrodes, such as plate electrodes. Recent studies suggest a potential solution to address the problem of field non-homogeneity in the tumor when plate electrodes are involved, i.e., delivering short (ns range) electric pulses at high frequencies (>MHz range). This approach is hypothesized to help alleviate the differences in dielectric properties within heterogeneous tissue (132) while ensuring saturated cell permeabilization (124). Other studies suggest to combine IRE and ECT procedures to facilitate the treatment and ensure complete tumor response (133), though in such case there is risk of thermal damage, and therefore the energy input should well controlled.

Patient-specific treatment planning has already been successfully used in radiotherapy, which, like electroporation, is also based on the interaction between a physical agent (radiation in radiotherapy and electric field in electroporation) and biological tissue (134). Radiotherapy consists of the following steps: simulation, treatment planning, setup verification, beam delivery, and response assessment. Simulation is based on the patient’s anatomy; the patient is scanned to obtain medical images [using, e.g., computed tomography (CT) or magnetic resonance imaging (MRI)] in the same position as expected to be when exposed to the radiation beam. Treatment planning starts by using the acquired medical images to generate a three-dimensional model. Similar approaches are being introduced in PEF-based treatments (135). In radiotherapy, the radiation dose must be high enough in the tumor volume to kill all the tumor cells, whereas in electrochemotherapy the electric field in the tumor volume needs to be sufficiently strong and the exposure long enough to cause cell membrane electroporation (33). Similarly to radiotherapy, the electrochemotherapy of deep-seated tumors can also be partitioned into several steps: mathematical modeling using CT and/or MRI images, treatment planning, setup verification, treatment, and response assessment.

## 8 Discussion

At the current state of scientific evidence, electrochemotherapy is a promising approach for various head neck cancers, allowing acceptable tumor response rates to be achieved. Nevertheless, our review revealed that complete or overall tumor response fluctuates across different cases. This can be attributed to various factors, including cancer type and selection of appropriate anti-cancer drug and its concertation as well as insufficient pre-treatment planning. To overcome these obstacles, treatment pre-planning should be better characterized and formalized as a compulsory protocol step. The standards for metrology of the pulses being delivered should also be improved. Finally, it should be highlighted that, currently, ECT in the context of H&N cancers is mostly used as a last resort when other treatment options are not viable. Based on the clinical data, it is indicatory that if ECT was used as a primary option for tumor treatment, the response rates should be significantly higher. The main limitation of ECT lies in the homogeneity of the spatial electric field distribution, which is dependent on electrode type and treated tissue heterogeneity. In case of non-accurate treatment pre-planning, the regrowth of the tumor is inevitable; however, if managed well, the PEF-based modality for cancer treatment offers very good response rates, non-toxic treatment, and fast procedure with little to no side effects. The flexibility to preserve aesthetics without compromises in treatment efficacy following the procedure could be attributed as one of the major motivators to use ECT in the context of head and neck cancers.

## Author contributions

AM: Formal analysis, Investigation, Methodology, Visualization, Writing – original draft, Writing – review & editing. VM: Investigation, Software, Validation, Visualization, Writing – original draft, Writing – review & editing. JK: Formal analysis, Investigation, Writing – original draft, Writing – review & editing. JI: Formal analysis, Investigation, Validation, Writing – original draft, Writing – review & editing. TI: Formal analysis, Investigation, Resources, Writing – original draft, Writing – review & editing. VN: Conceptualization, Formal analysis, Investigation, Methodology, Resources, Supervision, Validation, Writing – original draft, Writing – review & editing.
