# Supplementary material for: Electrochemotherapy for head and neck cancers: possibilities and limitations
Source: Front Oncol. 2024 Feb 15;14:1353800. doi: 10.3389/fonc.2024.1353800 (PMC10905418; doi:10.3389/fonc.2024.1353800)
Supplement: Supplementary file 1 [file DataSheet_1.pdf]

Supplementary Material

Author, year	Type of trial	Number of patients	Cancer type	Tumor size	Drug, dose, route	ECT parameters	Electrode type	Number of ECT sessions	Response measurement, evaluation time, results	Follow-up	Adverse events	Notes
Matthiessen LW et al. 2011 (1)	Phase II trial	Total:51 (H&N 30); Pre-treated: 42; Treatment-naive: 8.	ADC: 5; BC:15; BCC: 5; MM:21; SCC: 3; Other: 3.	≤3 cm: 138; >3 cm: 24.	Bleomycin (15000 IU/m^2); I.V: 30; I.T: 21.	Cliniporator™ (IGEA S.p.A., Italy); Pulses: 8; Duration: 100 µs; Amplitude: 1.3 kV/cm for plate electrodes; 1.0 kV/cm for needle electrodes; Frequency: 5 kHz	Plate; Hexagonal.	1	RECIST 1.0; Time: 2 months. OR: 79%; CR: 60%; PR: 19 %; SD: 11%; PD: 7%. N. a.: 3%. OR for ≤ 3 cm: 86% (CR: 68%); OR for > 3 cm: 31% (CR: 8%).	Median: 79 days (45 patients).	No SAE were observed. No grade 3 or 4 toxicity. Reported AE: Flu-like symptoms: 5; Pain in the treated area: 5; Ulceration of treated area:2; Cough:1; Allergic skin reaction:1; Anxiety:1.	30 H&N lesions were included in this study. Cancer type response n. s.
Gargiulo M et al. 2012 (2)	Retrospective	Total: 25.	ADC: 2 (pre-treated: 2); BCC: 9 (pre-treated: 0); Bowen: 1 (pre-treated: 1); SCC: 13 (pre-treated: 3).	N. s.	Bleomycin (15000 IU/m^2) I.V.	Based on ESOPE. Cliniporator™ (IGEA S.p.A., Italy); Pulses: 8; Duration: 100 µs; Amplitude: 1000 V/cm; Frequency: 5 kHz.	Hexagonal.	1-2	WHO guidelines; Time: 6 weeks. OR: 100%; CR: 72%; PR: 28%; OR (SCC):100%; CR (SCC): 65%; OR (BCC): 100%; CR (BCC): 100%.	Median: 18 months. No CR relapse during follow-up. 3 patients died of disease progression outside the treated area.	No SAE were reported. No hematological toxicity was reported. Local tumor necrosis: 2.	4 patients received surgery after ECT.
Mevio N et al. 2012 (3)	Retrospective	Total:15; Lesions: 31; Pre-treared:13; Treatment-naive:2.	BCC: 1 (pre-treated: 1); Merkel cell carcinoma:	N. s.	Bleomycin (15000 IU/m^2) I.V.	Based on ESOPE. Cliniporator™ (IGEA S.p.A., Italy).	Hexagonal; Finger.	1-3	RECIST 1.1; Time: 8 weeks. OR: 94%; CR:61.5%.	Median: 8.75 months (2-20). 1 died 3 days after	N. s.	Tumor response was evaluated according to the

			1 (pre-treated: 1); SCC: 13 (pre-treated: 11).						CR achieved in lesions <3 cm.	because of MI (excluded). 29% alive after 21 months.		assessable lesion.
Campana LG et al. 2014 (4)	Retrospective	Total:31; Lesions: 39; Treatment-naïve: 15. Pre-treated: 16.	ADC: 5; BCC: 9; SCC: 24.	Skin: ≤2 cm: 9; 2-4 cm: 13; >4 cm: 5; Median: 3.5 cm. Mucosal: ≤2 cm: 3; 2-4 cm: 7; >4 cm: 2; Median: 2.5 cm.	Cisplatin (0.5-2 mg/cm^3) I.T. Bleomycin (15000 IU/m^2) I.V.; (250-1000 IU/cm^3) I.T. I.V:7 I.T:7 I.V.+I.T.:25	Based on ESOPE. Cliniporator™ (IGEA S.p.A., Italy).	Finger; Plate; Linear; Hexagonal.	1-3	RECIST; Time: n. s. OR: 59%; CR: 38%.	Median: 14 months (3-82). 1-year overall LPFS: 59%.	No SAE reported. Confusion: 2; Facial oedema: 5; Median hospital stay:2 days (1-4).	Tumor site: Skin of the H&N: 27; Oral cavity and oropharynx: 12.

Landström FJ et al. 2015 (5)	Prospective	Toal:19; Treatment-naive: 19.	ADC:1; SCC:18.	N. s.	Bleomycin (1000 IU/cm^3) I.T.	Medpulser electroporation system (Inovio, San Diego, CA); Pulses: 6; Duration: 0.1 ms; Amplitude: 1100 V/cm.	N. s.	N. s.	Time: n. s. OR: 100%; CR:100%. 5-year local control in 12 surviving patients: 100%; 5-year tumor-specific survival: 75%.	Median: 58 months	No SAE reported. 1 patient with tongue base cancer developed aspiration.	All patients had mucosal (oral cavity or oropharynx). primary T1 or T2 cancer 2 patients received ND concurrent with ECT. 12 patients received RT following ECT. QoL data: significant increase in problems with senses, speech, mouth opening, xerostomia; decrease in problems with social contact.

Bertino G et al. 2016 (6)	Prospective	Total: 105;<br>Treatment-naive: 53;<br>Pre-treated: 52.	BCC: 34;<br>MM: 10;<br>SCC: 50;<br>Other: 11.	0.2-14.5 cm;<br>≤3 cm: 69;<br>>3 cm: 36.	Bleomycin;<br>I. V.: 97;<br>I. T: 8.	Based on ESOPE Cliniporator™ (IGEA S.p.A., Italy);<br>Electric pulses: 8;<br>Duration: 100 ms;<br>Amplitude: 1000 V/cm for needle electrodes; 1300 V/cm for plate electrodes.	Plate;<br>Hexagonal;<br>Finger.	1-2<br>2: 19 tumors.	RECIST 1.1 criteria;<br>Time: 2 months.<br>Total:<br>CR 62.6%;<br>PR 19.2%;<br>SD 13.1%;<br>PD 4.0%.<br>BCC:<br>CR 91%;<br>PR 6%;<br>SD 3%;<br>PD 0%.<br>SCC:<br>CR 55%;<br>PR 24%;<br>SD 15%<br>PD 4%.<br>MM:<br>CR 55%;<br>PR 22%;<br>SD 11%;<br>PD 11%.<br>Other:<br>CR 0%;<br>PR 44%;<br>SD 44%;<br>PD 11%.	1 year (median 6 months).<br>16 % of CR patients had recurrence after 8.3±3.5 months.<br>Overall survival rate 76%.	1 sepsis-related death post-treatment.<br>4 cases of tumor necrosis with loss of oral competence.	Multi-institutional study focused on skin cancers of H&N region.<br>QoL assessment:<br>EQ-5D: significant progressive positive perception of well-being.<br>QLQ-C30: significant improvement of physical functioning, decrease of fatigue and pain.<br>QLQ-H&N35: General improvement in all domains.
Guida M et al. 2016 (7)	Retrospective	Total: 19.	AGS:19.	1.5 - 3.5 cm;<br>Median: 2 cm.<br>.	Bleomycin (15000 IU/m^2)<br>I.V.	Based on ESOPE.<br>Cliniporator™ (IGEA S.p.A., Italy);<br>Pulses: 8;<br>Duration: 100 ms;<br>Frequency: 8 kHz.	Needle.	N. s.	Modified RECIST;<br>Time: 2 months.<br>Tumors:<br>OR: 85.5%;<br>CR:66.7%;<br>PR: 18.5%;<br>SD: 9.3%;<br>PD: 5.6%.<br>Patients:<br>OR: 63%;<br>CR:42%;<br>1-year DFS: 68%;<br>1-year PFS: 45%.	Median OS: 12 months (4-44).	No SAE during treatment of hospital stay.<br>Cutaneous toxicity: G1-6 patients;<br>G2-1 patient;<br>G3-2 patients.	This study focused on superficial AGS. 5 of 19 patients had scalp AGS.

Di Monta G et al. 2017 (8)	Retrospective	Total:22.	SCC:22.	N. s.	Bleomycin (15000 IU/m^2) I.V.	Based on ESOPE. Cliniporator™ (IGEA S.p.A., Italy).	Linear.	1-3<br>2: 7 patients;<br>3: 1 patient.	RECIST;<br>Time: 4 weeks.<br>OR: 81.8%;<br>CR: 22.7%;<br>PR: 59%;<br>SD:13,6%;<br>PD: 4.5%.	Median: 34 months (5-48)	Pain and erythema to the treated and surrounding area most common AE.	18 of 22 patients had skin cancer of the H&N.
Plaschke CC et al. 2017(9)	Phase II trial	Total:43.<br>Pre-treated: 43.	ACC:3;<br>ADC:1;<br>SCC:39.	Median: 3.5 cm.	Bleomycin (15000 IU/m^2) I.V: 2;<br>Bleomycin (1000 IU/ml) I.T: 41.	Based on ESOPE Cliniporator™ (IGEA S.p.A., Italy);<br>Pulses: 8;<br>Duration: 100 ms;<br>Amplitude: 1000 V/cm.	Linear;<br>Finger;<br>Hexagonal.	N. s.	RECIST 1.1;<br>Time: n. s.<br>Total:<br>OR: 56%;<br>CR:19%;<br>PR: 37%;<br>SD: 23%;<br>PD: 7%.<br><3cm OR:65%;<br>>3cm OR:50%	12 months.<br>1-year OS: 54%;<br>9 patients had no progression at the end of follow-up.	No SAE reported.	The study focused on mucosal H&N cancers.<br>37 of 43 patients were evaluable for tumor response.
Groselj A et al. 2017 (10)	Prospective	Total: 28.<br>Test group: 12 (24 lesions; 18 treatment-naive);<br>Control group: 16 (28 lesions; 24 treatment-naive).	Test group:<br>BCC: 17;<br>SCC: 7;<br>Control group:<br>BCC: 25;<br>SCC 3.	Test group:<br><2 cm: 12;<br>2-4 cm: 10;<br>>4 cm: 2.<br>Control group:<br><2 cm: 18;<br>2-4 cm: 8;<br>>4 cm: 2.	Control group: Bleomycin (15000 IU/m^2) I. V.<br>Test group: Bleomycin (10000 IU/m^2) I. V.	Based on ESOPE.	Hexagonal;<br>Linear;<br>Plate.	1	RECIST 1.1;<br>Time: 2 months.<br>Test group:<br>CR: 100% patients,<br>100 % tumors.<br>Control group:<br>CR: 94 % patients, 96 % tumors;<br>PR: 6 % patients, 4 % tumors.	2 months	Test group:<br>no AE related to treatment.<br>Control group: 2 patients (grade1, 2, 3).	Aim of the study – compare standard and reduced dose of bleomycin.
Gargiulo M et al. 2018 (11)	Retrospective	Total: 21.	SCC: 21.	N. s.	Bleomycin (15000 IU/m^2) I.V.	Based on ESOPE. Cliniporator™ (IGEA S.p.A., Italy);<br>Duration: 100 µs;<br>Amplitude: 1000 V/cm.	Linear.	1-2	WHO guidelines;<br>Time: 6 weeks.<br>OR: 100%;<br>CR: 71.4%.	Median:27 months.<br>No recurrent disease at 18-month follow-up: 85.7%.	No SAE reported.	The study focused on therapeutic, palliative or neoadjuvant treatment intent on patients with lower lip SCC.

Pichi B et al. 2019 (12)	Prospective	Total: 36; Pre-treated: 36.	ADC: 1; MM: 2; Sarcoma: 1; SCC: 31; Other: 1.	Palpable cervical metastases. Most lesions > 6 cm.	Bleomycin (15000 IU/m^2) I. V. 5 patients received adjunctive treatment.	Based on ESOPE.	Hexagonal; Finger; Linear.	1-4	RECIST 1.1; Time: 1 month. OR: 100 %; CR: 8.3%.	Mean: 7.6 months (2-18). 1-year OS: 41.6%; Median OS: 9 months.	No SAE reported. No post-operative bleeding events.	Study included mucosal and skin cancers of H&N region. Significant pain reduction after ECT. Mean VAS score 6.08 before treatment -> 1.25 1 month after treatment.
Plasche CC et al. 2019 (13)	Phase II trial	Total: 26; Pre-treated: 26.	ACC: 1; SCC: 25.	N. s. T1-T4	Bleomycin (15000 IU/m^2) I. V.	Besed on ESOPE. Cliniporator™ (IGEA S.p.A., Italy); Pulses: 8; Duration: 0.1 ms; Frequency: 1 or 5 kHz; Amplitude: 1 kV/cm.	Finger; Linear; Hexagonal.	1	RECIST 1.1; Time: 8 weeks. OR: 58 %.; CR: 19 %; PR: 39 %; SD: 35 % PD: 4 %. Biopsy 4 weeks after treatment: 6 out of 17 were without tumor. MRI: OR 82 %. PET/CT: SUV-peak reduction ≥30%: 76 %; SUV-peak progression ≥30%: 8 %; Stable metabolic disease: 16 %.	8 weeks. 2 patients were recurrence free 1 year after treatment.	CTCAE grade 4: 3 patients. CTCAE grade 3: 7 patients.	Study focused on mucosal cancers of H&N region. QoL assessment: EORTC QLQ-C30: no significant changes. EORTC QLQ-H&N35: “swallowing” and “social eating” changed significantly.

De Giorgi V et al. 2020 (14)	Prospective	Total: 8; Treatment-naïve: 5; Pre-treated: 3.	BCC: 4; SCC: 3; Non-melanoma skin metastasis: 1.	≤5 cm	Cisplatin (1 mg/ml) I.T.	Cliniporator™ (IGEA S.p.A., Italy). Duration: 100 ms; Amplitude: 1000 V/cm.	Linear; Lamellar; Finger.	1-2	Major tumor diameter, presence of tumor histologically. Time: N. s. OR: 100 %; CR: 50 %; PR: 50 %.	1 year. All tumors remained stable.	No severe toxicity was recorded. Local side effects 50% patients.	The study focused on skin cancers of H&N region.
Jamsek C et al. 2020 (15)	Prospective	Total: 28; Treatment-naïve: 28.	Standard dose (16 patients) BCC: 25; SCC: 3. Reduced dose (12 patients) BCC: 17; SCC: 7.	Standard dose: 0.4 cm-5.0 cm; Reduced dose: 0.6 cm-8.0 cm.	Bleomycin I.V.; Standard dose (15000 IU/m^2); Reduced dose (10000 IU/m^2).	N. s.	Hexagonal; Plate.	1	RECIST 1.1; Time: 2 months. Standard dose: CR 100%; Reduced dose: CR 96%.	Standard dose median: 40 months; Reduced dose median: 28 months. Tumor recurrence rate: Standard dose 15.4%; Reduced dose 39.0%.	N. s.	The study included treatment naïve patients >65 y.o. with non-melanoma skin cancer of H&N region.
Riva G et al. 2021(16)	Prospective	Total: 33 (27 evaluated).	ADC:5; BCC:4; SCC:18.	> 3 cm: 17 patients; < 3 cm: 10 patients.	Bleomycin (15000 IU/m^2) I.V.	Based on ESOPE; Cliniporator™ (IGEA S.p.A., Italy); Pulses: 8; Amplitude: 910-1000 V/cm; Frequency:5 kHz.	Linear; Finger.	N. s.	RECIST 1.1; Time: 1 month. OR: 48%; CR: 11%; PR: 37%; SD: 30%; PD: 22%.	1-6 months	No SAE reported. Bleeding control was achieved at T1 in all 7 patients who experienced it before ECT.	QoL

Sersa G et al. 2021(17)	Retrospective	<90 y.o. total:61 (41 H&N; 63 tumors);<br>>90 y.o. total:122 (44 H&N; 122 tumors).	>90 y.o.<br>BCC:16;<br>Breast Cancer:3;<br>MM:11;<br>SCC:28;<br>Other:3.<br><90 y.o.<br>BCC:32;<br>MM:22;<br>Breast cancer:6;<br>SCC:56;<br>Other:6.	>90 y.o.<br><3 cm: 122;<br>>3 cm: 33;<br>Median: 1.5 cm.<br><90 y.o<br><3 cm: 236;<br>>3 cm: 55;<br>Median: 1.5 cm.	Bleomycin I.V. or I.T.	Based on ESOPE.<br>Cliniporator™ (IGEA S.p.A., Italy);<br>Pulses: 8;<br>Amplitude: 910-1000 V/cm;<br>Duration: 100µs;<br>Frequency: 5 kHz.	Linear;<br>Plate;<br>Hexagonal;<br>Multiple.	1-2	Local response;<br>Time median: 38 days.<br>>90 y.o.<br>OR: 87;<br>CR: 57;<br>PR: 30;<br>SD: 8;<br>PD: 0;<br>NE: 5.<br><90 y.o.<br>OR: 88;<br>CR: 65;<br>PR: 23;<br>SD: 9;<br>PD: 2;<br>NE: 1.	>90 y.o.<br>median: 8 months (2-37).<br>Local reccurence: 8 (13%) patients.<br>< 90 y.o.<br>median: 5 months (0-46).<br>Local recurence: 11 (9%) patients.	No SEA reported.	This study focused of ECT use in older patients (>90 y.o.) with skin cancer.
Zimmermann G et al. 2021(18)	Prospective	Total: 21;<br>Test group: 9;<br>Control group: 12;<br>Treatment-naïve: 21.	SCC:21.	N. s.<br>T1-T2	Bleomycin (1 IU/cm^3) I.T.<br>Max dose 80 IU.	Medpulser electroporation system (Inovio, San Diego, CA);<br>Pulses: 6;<br>Amplitude: 1100 V/cm;<br>Duration: 0.1 ms.	Needle.	1	Resection of tumor and histological examination after 4-5 weeks.<br>Test group RD: 4;<br>Control group RD: 2.	Test group average: 67 months;<br>Control group average: 102 months.<br>OS: 71.4% (16/21);<br>Test group OS: 55.6%;<br>Control group OS: 91.6%.	Postoperative hemorrhage:<br>Test group 3/9;<br>Control group 1/12.<br>Pain:<br>Test group 77.7%;<br>Control group 25%;<br>Dysphagia:<br>Test group 66.6%;<br>Control group 30%.	This study compared ECT (test group) with surgery (control group) for early tongue cancer.

Claussen S et al 2022 (19)	Prospective	Total:716; Non-ulcerated lesion:414 (159 H&N); Ulcerated lesion:302 (155 H&N).	Non ulcerated BCC: 122; BC: 63; Kaposi sarcoma: 21; MM: 147; Sarcoma: 9; SPC: 33; Other: 19. Ulcerated BCC: 71; BC: 45; MM: 59; Sarcoma:6 ; Kaposi sarcoma:7; SPC: 96; Other:18.	Non-ulcerated Median: 1.5 cm. Ulcerated median: 30 mm.	Bleomycin (1000 IU/ml) I.T. Bleomycin (15000 IU/ m^2) I.V.	Based on ESOPE. Cliniporator™ (IGEA S.p.A., Italy); Pulses: 8; Duration: 100 µs.	Row; Hexagonal; Plate.	1	RECIST 1.0; Time: 1-2 months. Total OR: 83%; CR:59%; PR: 24%; SD: 13%. Non-ulcerated OR: 86%; CR:65%; PR: 21%; SD:11%; PD: 2%; NA: 1%. Ulcerated OR: 79%; CR:51%; PR: 28%; SD:15%; PD:4%; NE: 2%.	Minimum 180 days.	No SAE reported. Most common AE hyperpigmentation (22-29%).	The study focused on treatment outcomes for ulcerated and non-ulcerated cutaneous lesions. Data taken from InspECT.
Bertino G et al. 2022 (20)	Retrospective	Total: 330 (623 tumors; 496 H&N).	BCC	<3 cm: 560; >3 cm: 63.	Bleomycin (15000 IU/ m^2) I.V.: 184; Bleomycin (1000 IU/ml) I.T.: 146.	Based on ESOPE. Cliniporator™ (IGEA S.p.A., Italy); Pulses: 8; Amplitude: 400 V and 1000 V/cm (needle); 1300 V/cm (plate); Duration: 100 µs.	Row; Hexagonal; Plate.	1-2	RECIST 1.1; Time: 1 and 2 months. Per tumor OR: 96%; CR: 83.1%; PR: 12.9%; SD: 2.6%; PD: 0%; NA: 1.4 %. Per patient OR: 96%; CR: 80.7%; PR: 15.3%; SD: 3,0%; PD: 0%; NE: 1%.	In accordance with local institutional protocols. Overall 1- and 2-year LPFS: 96% and 90% .	No SAE reported.	The study focused on BCC treatment.

Bertino G et al. 2022 (21)	Retrospective	Total: 162 (342 tumors; 223 H&N).	SCC	≤3 cm: 241; ≥3cm: 101; Median: 2.1 cm.	Bleomycin; I.T.: 28; I.V.: 134.	Based on ESOPE. Cliniporator™ (IGEA S.p.A., Italy); Pulses: 8; Amplitude: 1000 V/cm; Duration: 100µs.	Hexagonal; Linear; Finger; Plate.	1-2	RECIST 1.1; Time: 45-90 days. OR: 79%; CR: 61%; PR: 18%; SD: 13%; PD: 5%.	Median: 5.6 months (1.6-47.6). Median time to local progression: 4.8 months; 1-year LPFS: primary patients 80%; locally advanced patients 49%.	SAE: 9 (4 %) Grade 4-5 AE not observed.	The study focused on SCC. Database was taken from InspECT.
Maglitto F et al. 2022 (22)	Retrospective	Total:101.	ADC:33; BCC:20; Epidermoid:6; SCC:72.	<2 cm: 7; 2-3 cm: 50; 3-4 cm: 41; 4-5 cm: 24; >5 cm: 9.	Bleomycin (15000 IU/m^2) I.V.	Based on ESOPE. Electroporator (IGEA srl, Carpi, Italy); Pulses: 8-96; Amplitude: 910-1000 V/cm; Duration: 100µs; Frequency: 5000 Hz.	Linear; Hexagonal; Finger.	N. s.	RECIST 1.1; Time: 2 months. OR: 40%; CR: 4%; PR: 36%; SD: 46%; PD: 14%.	N. s.	No SAE reported.	The aim of this study was to identify features involved in determining the partial response PR to ECT in patients with recurrent and/or metastatic H&N tumor.

Abbreviations

OR- overall response

CR- complete response

PR- partial response

SD- stable disease

PD- progressive disease

NE- inevaluable

ECT- electrochemotherapy

ND – neck dissection

RD- residual disease

H&N – head and neck

AE- adverse events

SAE – serious adverse events

MM- malignant melanoma

BC- breast cancer

ADC- adenocarcinoma

BCC- basal cell carcinoma

SCC- squamous cell carcinoma

SPC- spinocellular carcinoma

AGS- Angiosarcomas

LPFS- local progression-free survival

I.T. intratumorally

I.V. intravenously

AE- adverse events

N. s.- not specified

y.o. – years old

1. Matthiessen LW, Chalmers RL, Sainsbury DCG, Veeramani S, Kessell G, Humphreys AC, Bond JE, Muir T, Gehl J. Management of cutaneous metastases using electrochemotherapy. *Acta Oncol (Madr)* (2011) 50:621–629. doi: 10.3109/0284186X.2011.573626

2. Gargiulo M, Papa A, Capasso P, Moio M, Cubicciotti E, Parascandolo S. Electrochemotherapy for non-melanoma head and neck cancers: Clinical outcomes in 25 patients. *Ann Surg* (2012) 255:1158–1164. doi: 10.1097/SLA.0b013e31824f68b2

3. Mevio N, Bertino G, Occhini A, Scelsi D, Tagliabue M, Mura F, Benazzo M. Electrochemotherapy for the treatment of recurrent head and neck cancers: preliminary results.

4. Campana LG, Mali B, Sersa G, Valpione S, Giorgi CA, Strojan P, Miklavcic D, Rossi CR. Electrochemotherapy in non-melanoma head and neck cancers: A retrospective analysis of the treated cases. *British Journal of Oral and Maxillofacial Surgery* (2014) 52:957–964. doi: 10.1016/j.bjoms.2014.08.004

5. Landström FJ, Reizenstein J, Adamsson GB, Beckerath M Von, Möller C. Long-term follow-up in patients treated with curative electrochemotherapy for cancer in the oral cavity and oropharynx. *Acta Otolaryngol* (2015) 135:1070–1078. doi: 10.3109/00016489.2015.1049663

6. Bertino G, Sersa G, De Terlizzi F, Occhini A, Plaschke CC, Groselj A, Langdon C, Grau JJ, McCaul JA, Heuveling D, et al. European Research on Electrochemotherapy in Head and Neck Cancer (EURECA) project: Results of the treatment of skin cancer. *Eur J Cancer* (2016) 63:41–52. doi: 10.1016/j.ejca.2016.05.001

7. Guida M, Campana LG, Curatolo P, Strippoli S, Bonadies A, Grilz G, Cabula C, Rotunno R, Bucher S, Solari N, et al. Local treatment with electrochemotherapy of superficial angiosarcomas: Efficacy and safety results from a multi-institutional retrospective study. *J Surg Oncol* (2016) 114:246–253. doi: 10.1002/jso.24287

8. Di Monta G, Caracò C, Simeone E, Grimaldi AM, Marone U, Di Marzo M, Vanella V, Festino L, Palla M, Mori S, et al. Electrochemotherapy efficacy evaluation for treatment of locally advanced stage III cutaneous squamous cell carcinoma: A 22-cases retrospective analysis. *J Transl Med* (2017) 15: doi: 10.1186/s12967-017-1186-8

9. Plaschke CC, Bertino G, McCaul JA, Grau JJ, de Bree R, Sersa G, Occhini A, Groselj A, Langdon C, Heuveling DA, et al. European Research on Electrochemotherapy in Head and Neck Cancer (EURECA) project: Results from the treatment of mucosal cancers. *Eur J Cancer* (2017) 87:172–181. doi: 10.1016/j.ejca.2017.10.008

10. Groselj A, Bosnjak M, Strojan P, Krzan M, Cemazar M, Sersa G. Efficiency of electrochemotherapy with reduced bleomycin dose in the treatment of nonmelanoma head and neck skin cancer: Preliminary results. *Head Neck* (2018) 40:120–125. doi: 10.1002/hed.24991

11. Gargiulo M, Serra Mestre JM, Cortese A, Murphy DC, Parascandolo S, Razzano S. Long term effectiveness of electrochemotherapy for the treatment of lower lip squamous cell carcinoma. *Journal of Cranio-Maxillofacial Surgery* (2018) 46:1968–1974. doi: 10.1016/j.jcms.2018.08.013

12. Pichi B, Pellini R, Spriano G. Electrochemotherapy – A locoregional therapy with well-established palliative effect in patient with large recurrent lesion of head and neck. *Journal of Cranio-Maxillofacial Surgery* (2019) 47:41–46. doi: 10.1016/j.jcms.2018.10.014

13. Plaschke CC, Johannesen HH, Hansen RH, Hendel HW, Kiss K, Gehl J, Wessel I. The DAHANCA 32 study: Electrochemotherapy for recurrent mucosal head and neck cancer. *Head Neck* (2019) 41:329–339. doi: 10.1002/hed.25454

14. De Giorgi V, Scarfì F, Saqer E, Gori A, Tomassini GM, Covarelli P. The use of cisplatin electrochemotherapy in nonmelanoma skin cancers: A single-center study. *Dermatol Ther* (2020) 33: doi: 10.1111/dth.13547

15. Jamsek C, Sersa G, Bosnjak M, Groselj A. Long term response of electrochemotherapy with reduced dose of bleomycin in elderly patients with head and neck non-melanoma skin cancer. *Radiol Oncol* (2020) 54:79–85. doi: 10.2478/raon-2020-0009

16. Riva G, Salonia L, Fassone E, Sapino S, Piano F, Pecorari G. Quality of life in electrochemotherapy for cutaneous and mucosal head and neck tumors. *J Clin Med* (2021) 10: doi: 10.3390/jcm10194366

17. Sersa G, Mascherini M, Di Prata C, Odili J, de Terlizzi F, McKenzie GAG, Clover AJP, Bertino G, Spina R, Groselj A, et al. Outcomes of older adults aged 90 and over with cutaneous malignancies after electrochemotherapy with bleomycin: A matched cohort analysis from the InspECT registry. *European Journal of Surgical Oncology* (2021) 47:902–912. doi: 10.1016/j.ejso.2020.10.037

18. Zimmermann CE, Faesser HA, Gassling V, Wiltfang J. The role of electrochemotherapy with intratumoral bleomycin for early tongue carcinoma. *Acta Otolaryngol* (2021) 141:424–431. doi: 10.1080/00016489.2020.1871511

19. Claussen CS, Moir G, Bechara FG, Orlando A, Matteucci P, Mowatt D, Clover AJP, Mascherini M, Gehl J, Muir T, et al. Prospective cohort study by InspECT on safety and efficacy of electrochemotherapy for cutaneous tumors and metastases depending on ulceration. *JDDG - Journal of the German Society of Dermatology* (2022) 20:470–481. doi: 10.1111/ddg.14699

20. Bertino G, Groselj A, Campana LG, Kunte C, Schepler H, Gehl J, Muir T, Clover JAP, Quaglino P, Kis E, et al. Electrochemotherapy for the treatment of cutaneous squamous cell carcinoma: The INSPECT experience (2008-2020). *Front Oncol* (2022) 12: doi: 10.3389/fonc.2022.951662

21. Bertino G, Muir T, Odili J, Groselj A, Marconato R, Curatolo P, Kis E, Lonkvist CK, Clover J, Quaglino P, et al. Treatment of Basal Cell Carcinoma with Electrochemotherapy: Insights from the InspECT Registry (2008–2019). *Current Oncology* (2022) 29:5324–5337. doi: 10.3390/curroncol29080423

22. Maglitto F, Salzano G, Longo F, Di Bernardo E, D'alessio V, Fusco R, Aversa C, Pavone E, Pontone M, Marciano ML, et al. Electrochemotherapy as palliative treatment in patients with recurrent and/or metastatic head and neck tumours: features analysis for an early determination of the partial responsive patients. (2022). 1350–1363 p.
